# Hsa-let-7c-5p, hsa-miR-130b-3p, and hsa-miR-142-3p as Novel miRNA Biomarkers for Melanoma Progression

**DOI:** 10.1155/2022/5671562

**Published:** 2022-07-07

**Authors:** Xuerui Wu, Yu Wang, Chen Chen, Yadong Xue, Shuyun Zheng, Limin Cai

**Affiliations:** Department of Dermatology, The First Affiliated Hospital of Harbin Medical University, Harbin, Heilongjiang 150001, China

## Abstract

This study aimed to screen miRNA biomarkers for melanoma progression. Raw melanoma data were downloaded from the Gene Expression Omnibus (GSE34460, GSE35579, GSE18509, and GSE24996) and the Cancer Genome Atlas (TCGA). Then, all differentially expressed miRNAs (DEmiRNAs) between benign vs. primary, metastatic vs. benign, and metastatic vs. primary groups were obtained in the GSE34460 and GSE35579 datasets, and the miRNAs related to disease progression were further screened. Then, the miRNA-gene network was constructed, followed by enrichment, survival, and cluster analyses. Differentially expressed genes (DEGs), tumor-infiltrating immune cells, and tumor mutation burden (TMB) between subtypes were analyzed. miRNAs were verified in the GSE18509 and GSE24996 datasets. A total of 132 and 209 DEmiRNAs were obtained in the GSE34460 and GSE35579 datasets, respectively, and 27 DEmiRNAs related to disease progression were screened. hsa-miR-106b-5p, hsa-miR-27b-3p, and hsa-miR-141-3p had a higher degree and were regulated by numerous genes in the miRNA-gene network. Moreover, four miRNAs were associated with prognosis: hsa-let-7c-5p, hsa-miR-130b-3p, hsa-miR-142-3p, and hsa-miR-509-3p. Furthermore, the bidirectional hierarchical clustering of 27 miRNAs was classified into three subtypes, and TMB and four types of immune cells, including activated dendritic cells, naïve CD4 T cells, M1 macrophages, and plasma cells, showed significant differences among the three subtypes. The expression levels of most miRNAs in the GSE18509 and GSE24996 datasets were consistent with those in the training dataset. These miRNAs, including hsa-let-7c-5p, hsa-miR-130b-3p, and hsa-miR-142-3p, and activated dendritic cells, naïve CD4 T cells, M1 macrophages, and plasma cells may play vital roles in the pathogenesis of melanoma.

## 1. Introduction

Melanoma is a highly aggressive malignant tumor originating from neural crest melanocytes [1, 2]. Melanoma cells can infiltrate the tissues, lymph, and blood vessels. Melanoma accounts for 3% of all malignant tumors of the skin [3], with its incidence rate increasing rapidly worldwide [4, 5]. In 2017, the incidence rate of melanoma in China was 0.9/10 million by age [6]. Melanoma is not sensitive to radiotherapy, chemotherapy, and biological immunotherapy, and its invasiveness and early metastasis make it the deadliest among skin malignant tumors, with only a 6% five-year survival rate of stage IV patients [7]. Thus, it is necessary to screen for reliable and effective biomarkers for the diagnosis and prognosis of melanoma.

MicroRNAs (miRNAs) are gene regulatory factors with excellent functions, ranging in size from 19 to 25 nucleotides [8]. miRNAs participate in the posttranscriptional control of gene expression by binding to the 3′-untranslated region (3′-UTR) of the target mRNA and then inhibits or degrades the target gene [9]. This molecular-level imbalance in biological processes is a potential mechanism for the development of many diseases. Numerous miRNAs have been reportedly involved in melanoma development. Lu et al. found that five miRNAs, including miR-25, miR-204, miR-211, miR-510, and miR-513c, could be used as prognostic biomarkers for patients with cutaneous melanoma cancer [10]. Zhu et al. revealed that miRNA-378a-3p, miRNA-23b-3p, and miRNA-200c-3p are associated with the occurrence of skin cutaneous melanoma [11]. Li et al. suggested that miR-300 and miR-629 are associated with melanoma [12]. However, these studies screened the miRNAs related to melanoma based on a single dataset with a small sample size, and few studies have identified the miRNAs involved in the progression of melanoma. Therefore, miRNA biomarkers for melanoma progression should be identified based on multiple datasets.

**Figure 1:**
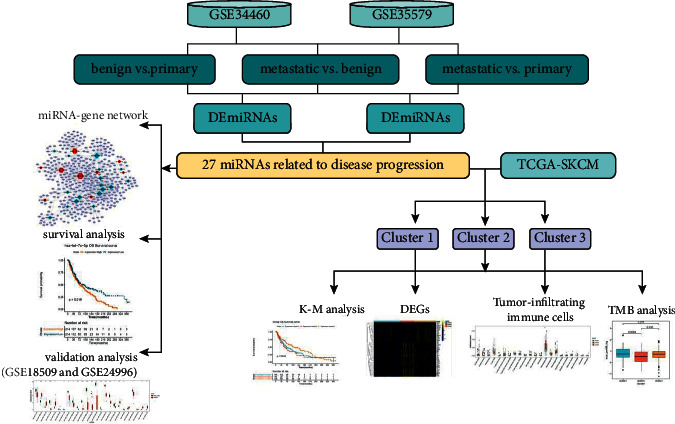
The flowchart of this study.

This study aimed to screen miRNA biomarkers for melanoma progression. Using the data downloaded from the Gene Expression Omnibus (GEO) and the Cancer Genome Atlas (TCGA) databases, miRNAs related to melanoma progression were obtained, the miRNA-gene network was constructed, and prognosis-related miRNAs were screened. Moreover, cluster analysis was conducted based on miRNAs related to melanoma progression, and the differentially expressed genes (DEGs), tumor-infiltrating immune cells, and tumor mutation burden (TMB) between subtypes were analyzed. This study provided a powerful basis for identifying novel therapeutic targets for melanoma patients. A flowchart of this study is shown in Figure 1.

## 2. Materials and Methods

### 2.1. Data Collection

Four datasets that included some patients with melanoma were included from the GEO database [13], including GSE34460 (8 benign melanoma, 9 primary melanoma, and 4 metastatic melanoma, detection platform: GPL15019 Agilent-031181 Unrestricted_Human_miRNA_V16.0_Microarray 030840 (miRBase release 14.0 miRNA ID version)), GSE35579 (11 benign melanoma, 20 primary melanoma, and 21 metastatic melanoma, detection platform: GPL15183 CRUK/Melton lab-Human melanoma-71-v2-microRNA expression profiling), GSE18509 (8 benign melanoma, 8 metastatic melanoma, detection platform: GPL9081 Agilent-016436 Human miRNA Microarray 1.0 G4472A (miRNA ID version)), and GSE24996 (8 benign melanoma, 15 primary melanoma, and 8 metastatic melanoma, detection platform: GPL6955 Agilent-016436 Human miRNA Microarray 1.0 (feature number version)). Moreover, the miRNA mature strand expression RNAseq data (log2 (RPM+1)), gene expression RNAseq data (log2 (norm_count+1)), and the survival information of the corresponding samples (overall survival (OS), disease-specific survival (DSS), and progression-free interval (PFI)) of skin melanoma (SKCM) were obtained from TCGA of UCSC-Xena [14]. The annotated MAF file processed by the MuTect software of somatic mutation data (SNPs and small INDELs) of TCGA-SKCM was obtained from the Genomic Data Commons (GDC) database (https://portal.gdc.cancer.gov) for subsequent mutation correlation analysis.

### 2.2. Data Processing

For the GSE34460, GSE18509, and GSE24996 datasets, the raw data were preprocessed using limma [15] in the *R* package. The data preprocessing processes included data reading, RMA background adjustment, quantile normalization, and normalization. The expression matrix of probes was obtained, and the probes were annotated to miRNA ID according to the platform annotation information; then, the miRNA ID was converted using miRBase website [16], and the obtained miRNAs were used for the subsequent analysis. For the GSE35579 dataset, the processed probe expression matrix was downloaded, which was log-transformed, and the probes were annotated to miRNA ID according to the platform annotation information. Then, the miRNA ID was converted using miRBase website, and the obtained miRNAs were used for the subsequent analysis. For the TCGA-SKCM dataset, samples with an OS. time > 0 were selected for subsequent cluster analysis.

### 2.3. Differential Expression Analysis

Based on the GSE34460 and GSE35579 datasets, the typical Bayesian test provided by the *R* package limma package was used to screen the differentially expressed miRNAs (DEmiRNAs) between benign vs. primary, metastatic vs. benign, and metastatic vs. primary, with a cutoff value of *P* < 0.05 and |logFC| > 0.5. Then, all overlapping DEmiRNAs screened between benign vs. primary, metastatic vs. benign, and metastatic vs. primary were obtained in each dataset, and the common DEmiRNAs were obtained from the two datasets. Based on the common DEmiRNAs, the expressed values of the common DEmiRNAs in each sample of the GSE34460 and GSE35579 datasets were extracted. For the same group of samples, the average of each miRNA was calculated as the average level of the miRNA in the sample group. After comparing the expression levels of DEmiRNAs in the benign, primary, and metastatic groups, the DEmiRNAs were divided into four types: expression continuous upregulation (up-up), expression continuous downregulation (down-down), upregulated first and then downregulated (up-down), and downregulated first and then upregulated (down-up). The miRNAs that overlapped in the same types were considered miRNAs related to disease progression for subsequent analysis.

### 2.4. Prediction of Target Genes of miRNA and Enrichment Analysis

Based on the six databases, miRWalk, Microt4, miRanda, miRDB, RNA22, and TargetScan, the miRNA-gene interaction pairs were screened using the miRWalk 2.0 tool [17]. The miRNA-gene interaction pairs overlapped in the six databases were identified. Moreover, the “Skin Cutaneous Melanoma” was used as the keyword to screen the disease-related genes in the GeneCards database [18]. The disease-related genes were intersected with target genes of miRNA that overlapped in the six databases, and the miRNA-gene interaction pairs were further obtained, followed by visualization using Cytoscape [19]. Additionally, clusterProfiler [20] in the *R* package was used to perform enrichment analysis of the genes. The *P* value was corrected using Benjamini and Hochberg (BH), and the adjust *P* < 0.05 was regarded as a statistically significant difference.

### 2.5. Survival Analysis

Based on the miRNAs related to disease progression, the expression values of the miRNAs in each sample were extracted from the miRNA expression data in TCGA-SKCM samples. The patients were then allocated to high and low-miRNA expression groups according to the median expression value, and the Kaplan–Meier curve method was used to evaluate the association between miRNAs and prognosis.

### 2.6. Cluster Analysis of miRNAs

Based on the miRNAs related to disease progression and the expression value of miRNAs in each sample in TCGA-SKCM, bidirectional hierarchical clustering was conducted based on the centered Pearson correlation algorithm using pheatmap [21] to identify different subtypes. The Kaplan–Meier curve method was used to evaluate the association between subtypes and prognosis.

### 2.7. Identification of DEGs between Subtypes

The gene expression matrix corresponding to each subtype sample was extracted, and the DEGs were identified between the two subtypes using limma in the *R* package. The *P* value was corrected using BH, and the adjust *P* < 0.05 and |logFC| > 0.5 were set as the cutoff values. Moreover, the DAVID tool [22] was used to perform enrichment analysis on the common DEGs with a threshold value of *P* < 0.05 and count ≥ 2.

### 2.8. Tumor-Infiltrating Immune Cells

Based on the gene expression level of samples in each subtype, the CIBERSORT algorithm [23] was utilized to evaluate the proportion of immune cells in subtypes, and the differences in the proportion of immune cells in subtypes were analyzed using the Kruskal–Wallis test with a threshold value of *P* < 0.05.

### 2.9. TMB Analysis

The Oncoplot function in Maftools [24] was used to visualize the top 20 genes with the highest mutation frequency in each subtype based on the genetic mutation information in each sample. The TMB value was calculated, and the differences in TMB values in subtypes were analyzed using the *t*-test with a threshold value of *P* < 0.05.

### 2.10. Validation Analysis

To verify that the miRNAs were related to melanoma, the GSE18509 and GSE24996 datasets were used as the validation datasets. The expression values of miRNAs in each sample in the GSE18509 and GSE24996 datasets were extracted, and the differences in expression levels between benign and metastatic groups were analyzed using the *t*-test.

## 3. Results

### 3.1. Identification of DEmiRNAs

**Figure 2:**
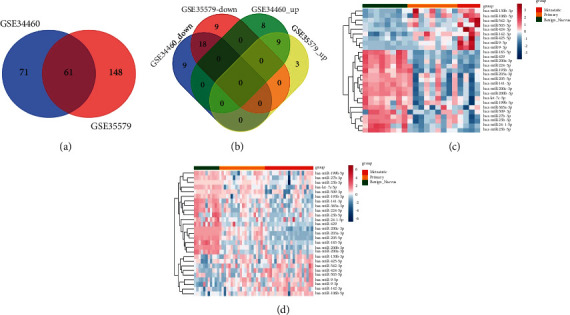
Identification of differentially expressed miRNAs (DEmiRNAs) related to melanoma progression. (a) The common DEmiRNAs screened in GSE34460 and GSE35579 datasets. (b) The miRNAs related to melanoma progression. The heatmaps of the miRNAs related to melanoma progression in GSE34460 (c) and GSE35579 (d) datasets, respectively.

Based on the GSE34460 and GSE35579 datasets, DEmiRNAs between benign vs. primary, metastatic vs. benign, and metastatic vs. primary were identified. As given in Table 1, 80, 107, and 24 DEmiRNAs were obtained between benign vs. primary, metastatic vs. benign, and metastatic vs. primary in the GSE34460 dataset, respectively, and 138, 127, and 78 DEmiRNAs were screened between benign vs. primary, metastatic vs. benign, and metastatic vs. primary in the GSE35579 dataset, respectively. Thus, a total of 132 and 209 overlapping DEmiRNAs were obtained in the GSE34460 and GSE35579 datasets, respectively. Then, totally 61 common DEmiRNAs were screened (Figure 2(a)). A total of 27 DEmiRNAs that overlapped in the same types were considered as miRNAs related to disease progression, including 18 and 9 miRNAs in down-down- and up-up types, respectively (Figure 2(b)). Heatmaps of the 27 DEmiRNAs in the GSE34460 and GSE35579 datasets are shown in Figures 2(c) and 2(d), respectively.

### 3.2. Prediction of Target Genes of miRNA and Enrichment Analysis

**Figure 3:**
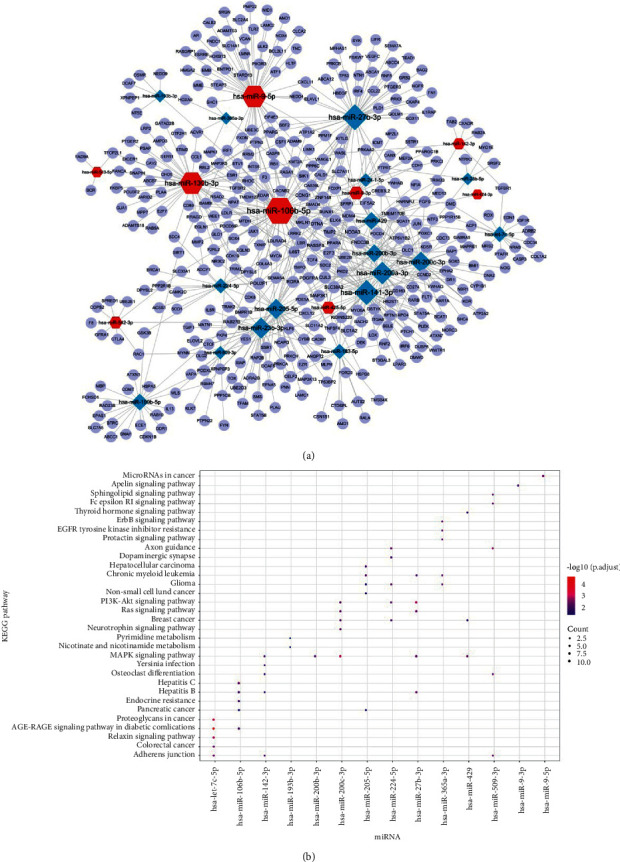
Prediction of target genes of miRNA and enrichment analysis. (a) The miRNA-gene network. (b) The results of the Kyoto Encyclopedia of Genes and Genomes (KEGG) pathway enrichment analysis. The size of the ball represents the number of genes enriched in each term. The color of the ball represents the *P* value.

A total of 2330 miRNA-gene interaction pairs containing 26 miRNAs and 1490 genes were screened using miRWalk 2.0. Then, the disease-related genes screened in the GeneCards database were intersected with target genes of miRNA, and a total of 604 miRNA-gene interaction pairs were further obtained, including 26 miRNAs and 377 genes. The miRNA-gene network is shown in Figure 3(a). hsa-miR-106b-5p, hsa-miR-27b-3p, and hsa-miR-141-3p had higher degree and were regulated by numerous genes (Table 2). The Kyoto Encyclopedia of Genes and Genomes (KEGG) pathway enrichment analysis showed that the target genes of 14 miRNAs were involved in the MAPK and PI3K-Akt signaling pathways, and the top5 enriched pathways are shown in Figure 3(b).

### 3.3. Survival Analysis

**Figure 4:**
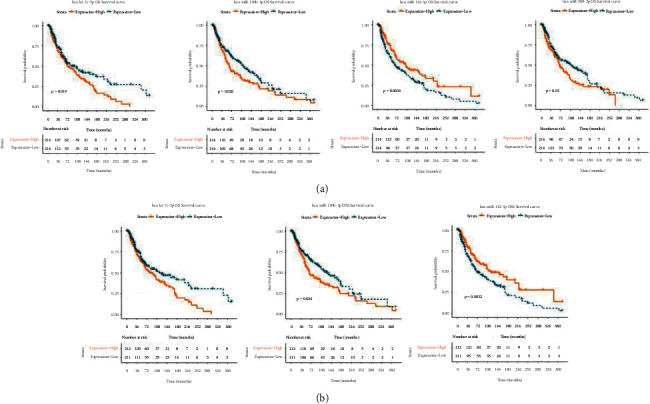
Survival analysis. (a) Four miRNAs associated with overall survival (OS). (b) Three miRNAs correlated with disease-specific survival (DSS).

To evaluate the relationship between the 27 miRNAs and prognosis, survival analysis was conducted using the Kaplan–Meier curve method, and the results showed that four miRNAs were associated with OS (hsa-let-7c-5p, hsa-miR-130b-3p, hsa-miR-142-3p, and hsa-miR-509-3p) (all *P* < 0.05, Figure 4(a)), and three miRNAs were correlated with DSS (hsa-let-7c-5p, hsa-miR-130b-3p, and hsa-miR-142-3p) (all *P* < 0.05, Figure 4(b)); however, there were no miRNAs related to PFI.

### 3.4. Cluster Analysis of miRNAs

**Figure 5:**
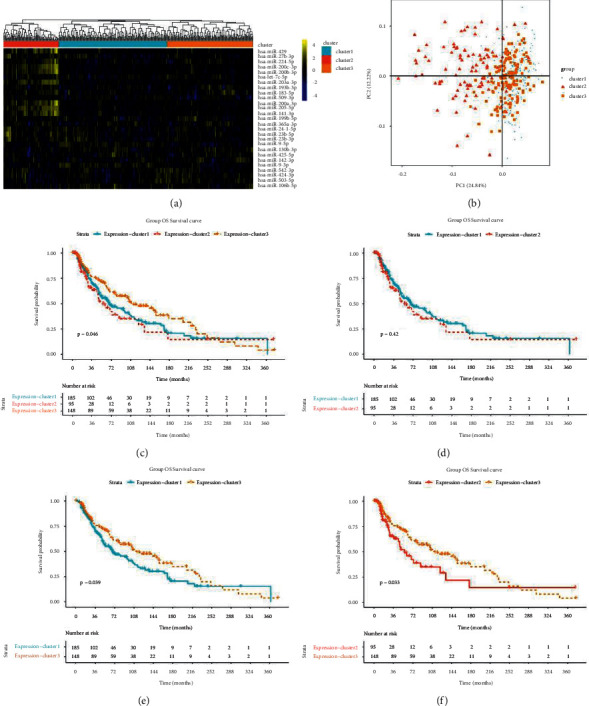
Cluster analysis of miRNAs. (a) The 27 miRNAs classified into three subtypes (clusters 1, 2, and 3). (b) The principal component analysis (PCA) of three subtypes. Kaplan–Meier curve of the three subtypes (c), cluster 1 and 2 (d), cluster 1 and 3 (e), and cluster 2 and 3 (f).

Bidirectional hierarchical clustering was conducted based on the expression value of the 27 miRNAs in each sample in TCGA-SKCM. As shown in Figure 5(a), the bidirectional hierarchical clustering of the 27 miRNAs was classified into three subtypes (clusters 1, 2, and 3), and most miRNAs were highly expressed in cluster 2; however, miRNAs showed low expression in clusters 1 and 3. The principal component analysis (PCA) of three subtypes is shown in Figure 5(b). Moreover, survival analysis showed that clusters 1 and 2 were associated with poor OS, and cluster 3 correlated with better OS (Figure 5(c)). Moreover, the Kaplan–Meier curve was drawn between the two clusters, and the results showed that clusters 3 and 1 and clusters 3 and 2 had significant differences (all *P* < 0.05, Figures 5(d) and 5(e)). There was no significant difference between clusters 1 and 2 (*P* < 0.05, Figure 5(f)).

### 3.5. Identification of DEGs between Subtypes

**Figure 6:**
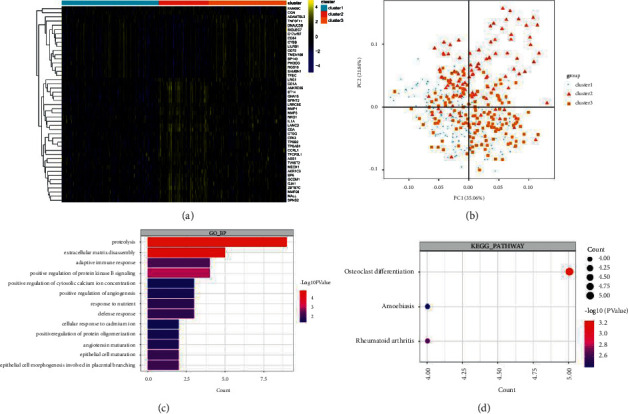
Identification of differentially expressed genes (DEGs) between subtypes. (a) The heatmap of 47 common DEGs. (b) The principal component analysis (PCA) of 47 common DEGs. (c) The gene ontology (GO) enrichment analysis. Length of bar chart represents the number of genes enriched in each term. The color of the ball represents the *P* value. (d) The Kyoto Encyclopedia of Genes and Genomes (KEGG) pathway enrichment analysis. The size of the ball represents the number of genes enriched in each term. The color of the ball represents the *P* value.

A total of 1846, 679, and 2257 DEGs were identified between clusters 1 and 2, clusters 1 and 3, and clusters 2 and 3, respectively, and 47 common DEGs were identified (Figure 6(a)). PCA of the 47 common DEGs in clusters 1, 2, and 3 is shown in Figure 6(b). Moreover, enrichment analysis was conducted on 47 common DEGs. These were enriched in 13 gene ontology (GO)-biology process (BP) (Figure 6(c)) and three KEGG pathways (osteoclast differentiation, amoebiasis, and rheumatoid arthritis) (Figure 6(d)).

### 3.6. Tumor-Infiltrating Immune Cells

**Figure 7:**
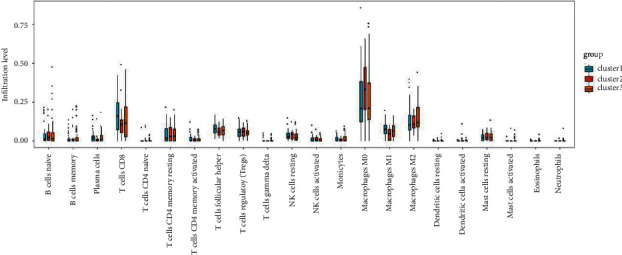
The proportion of 22 subtypes of immune cells.

The CIBERSORT algorithm was used to evaluate the proportion of subtypes of immune cells, and the differences in these proportions were analyzed using the Kruskal–Wallis test. The results showed significant differences in four subtypes of immune cells, including activated dendritic cells (*P* < 0.05), naïve CD4 T cells (*P* < 0.05), M1 macrophages (*P*=0.02), and plasma cells (*P*=0.02). A box diagram of the proportion of 22 subtypes of immune cells is shown in Figure 7.

### 3.7. TMB Analysis

**Figure 8:**
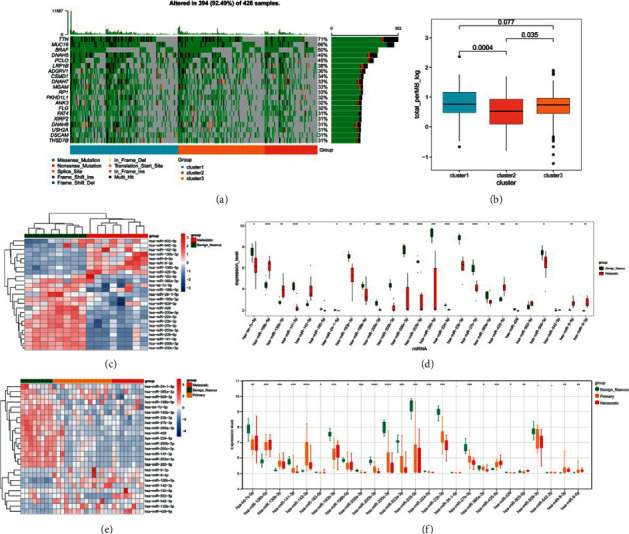
Tumor mutation burden (TMB) analysis and validation analysis. (a) The waterfall plot of the top 20 genes with the highest mutation frequency in each subtype. (b) the TMB value in subtypes. The heatmap (c) and box diagram (d) of 25 miRNAs between samples from benign and metastatic groups in the GSE18509 dataset. The heatmap (e) and box diagram (f) of 25 miRNAs between samples from benign and metastatic groups in the GSE24996 dataset. ^*∗*^*P* < 0.05.

The waterfall plot of the top 20 genes with the highest mutation frequency in each subtype is shown in Figure 8(a), which revealed that the gene mutation frequency in cluster 2 was low, the gene mutation frequency in cluster 1 was high, and *TTN*, *MUC16*, *BRAF*, and *DNAH5* in the three subtypes had high mutation frequencies. In addition, the differences in TMB values in subtypes were analyzed, and the TMB value in cluster 2 was significantly lower than that in the other two subtypes (*P* < 0.05) (Figure 8(b)).

### 3.8. Validation Analysis

The GSE18509 and GSE24996 datasets were used as validation datasets to verify that the 27 miRNAs were related to melanoma. As none of the probes in the GSE18509 dataset matched hsa-miR-424-3p and hsa-miR-23b-5p, 25 miRNAs were verified. The heatmap showed that 25 miRNAs were well distinguished between samples from the benign and metastatic groups (Figure 8(c)), suggesting that 25 miRNAs were related to melanoma. The differences in expression levels between benign and metastatic groups were evaluated using the *t*-test, and the box diagram showed that the expression levels of most miRNAs were significantly different, and the regulation trend was consistent with the results of the training dataset (Figure 8(d)). Moreover, the expression of 27 DEmiRNAs was analyzed using the GSE24996 dataset. Because the two probes in the GSE24996 dataset were not matched, 25 miRNAs were verified. The results showed that the expression of 22 DEmiRNAs was significantly different among the benign, primary, and metastatic groups (Figures 8(e) and 8(f)), suggesting that the above results were reliable.

## 4. Discussion

miRNAs are aberrantly expressed in numerous cancers [25], including melanoma. This study aimed to screen miRNA biomarkers of melanoma using data downloaded from public databases. hsa-miR-106b-5p, hsa-miR-27b-3p, and hsa-miR-141-3p had a higher degree and were regulated by numerous genes in the miRNA-gene network, and the target genes of miRNAs were involved in the MAPK and PI3K-Akt signaling pathways. Moreover, four miRNAs were associated with prognosis: hsa-let-7c-5p, hsa-miR-130b-3p, hsa-miR-142-3p, and hsa-miR-509-3p. Moreover, the bidirectional hierarchical clustering of the 27 miRNAs was classified into three subtypes, and TMB and four subtypes of immune cells, including activated dendritic cells, naïve CD4 T cells, M1 macrophages, and plasma cells, showed significant differences among the three subtypes.

Sixty-one common DEmiRNAs were screened between benign vs. primary, metastatic vs. benign, and metastatic vs. primary in the GSE34460 and GSE35579 datasets. Among these, 27 DEmiRNAs were related to disease progression. Moreover, the miRNA-gene network was constructed, and hsa-miR-106b-5p, hsa-miR-27b-3p, and hsa-miR-141-3p had a higher degree and were regulated by numerous genes. Chen et al. found that hsa-miR-106-5p promotes the cell cycle progression of malignant melanoma by targeting PTEN [26]. Bonnu et al. revealed that hsa-miR-106b-5p regulates prostate cancer by interfering with the endoplasmic reticulum stress repair pathway and reducing the expression of tumor suppressor genes involved in many biological processes [27]. Liu et al. suggested that hsa-miR-106b-5p promotes colorectal cancer cell migration and invasion [28]. Gencia et al. found that hsa-miR-27b-3p showed altered expression in all three melanoma types [29]. Miao et al. indicated that hsa-miR-27b-3p inhibits glioma development by targeting YAP1 [30]. Verrando et al. found that transnonachlor reduces the level of hsa-miR-141-3p in human melanocytes in vitro and promotes the characteristics of melanoma cells [31]. In addition, the KEGG pathway enrichment analysis showed that the target genes of 14 miRNAs were involved in the MAPK and PI3K-Akt signaling pathways. Chen et al. illustrated that FARP1 promotes cell proliferation by regulating the MAPK signaling pathway in cutaneous melanoma [32]. Wu et al. found that lncRNA OR3A4 facilitates the invasion and migration of melanoma cells through the PI3K/Akt signaling pathway [33]. Thus, miRNAs may be involved in the progression of melanoma via the MAPK and PI3K-Akt signaling pathways.

Three miRNAs, hsa-let-7c-5p, hsa-miR-130b-3p, and hsa-miR-142-3p, were related to both OS and DFF, and hsa-miR-509-3p was associated with OS. Liu et al. found that hsa-let-7c-5p was associated with melanoma metastasis [34]. Fu et al. suggested that hsa-let-7c-5p restrains breast cancer cell proliferation and facilitates apoptosis by targeting ERCC6 [35]. Murria et al. identified miRNAs associated with melanoma survival, and the results suggested poorer survival in melanomas with hsa-miR-130b-3p overexpression [36]. Tembe et al. indicated that hsa-miR-142-3p is related with the prognosis of patients with metastatic melanoma [37]. Li et al. found that GPC6 is an early biomarker for metastatic progression of melanoma, which can be regulated by hsa-miR-509-3p [38]. Patil et al. revealed that hsa-miR-509-3p inhibits the migration, invasion, and proliferation of osteosarcoma cells [39]. Our results were in line with those reported in previous studies, suggesting that hsa-let-7c-5p, hsa-miR-130b-3p, hsa-miR-142-3p, and hsa-miR-509-3p are associated with the prognosis of patients with melanoma.

Tumor-infiltrating immune cells play a vital role in promoting or inhibiting tumor growth [40]. The bidirectional hierarchical clustering of the 27 miRNAs was classified into three subtypes, and four subtypes of immune cells, including activated dendritic cells, naïve CD4 T cells, M1 macrophages, and plasma cells, showed significant differences among the three subtypes. Among the immune cells involved in cancer immunity, properly activated plasmacytoid dendritic cells play a vital role in building a bridge between inherent and adaptive immune responses and directly eliminating cancer cells [41]. Dendritic cells with potent antigen-presenting abilities have long been considered a critical factor in antitumor immunity [42]. Su et al. found that the abundance of naïve CD4^+^ T cells is closely related to Tregs, suggesting that the prognosis of breast cancer patients is poor [43]. Tumor-associated macrophages could be used as treatment targets in oncology [44, 45]. Plasma cells are the main effectors of adaptive immunity and are responsible for producing antibodies to protect the body against pathogens. Numerous studies have reported that plasma cells are related to the pathogenesis of cancer, such as triple-negative breast cancer [46], lung cancer [47], and colorectal cancer [48]. Moreover, TMB could be considered an immunotherapy biomarker for cancer [49–51]. In this study, the TMB value in cluster 2 was significantly lower than that in the other two subtypes, suggesting that these miRNAs might play important roles in the pathogenesis of melanoma by involving tumor-infiltrating immune cells and TMB.

However, this study has certain limitations. First, the data analyzed in this study were downloaded from public databases, and external validation is required. Moreover, the sample size should be expanded in future studies. Relevant experiments must be performed to verify the miRNAs identified in our bioinformatics analyses.

## 5. Conclusions

In summary, this study identified numerous novel miRNA biomarkers, including hsa-miR-106b-5p, hsa-miR-27b-3p, and hsa-miR-141-3p, which play important roles in melanoma and might provide new insights into the pathogenesis of melanoma.

## Figures and Tables

**Table 1:** Identification of differentially expressed miRNAs (DEmiRNAs) in GSE34460 and GSE35579 datasets.

Dataset	Group	Upregulation	Downregulation	Total
GSE34460	Primary–Benign_Naevus	26	54	80
	Metastatic–Benign_Naevus	51	56	107
	Metastatic–primary	18	6	24

GSE35579	Primary–Benign_Naevus	52	86	138
	Metastatic–Benign_Naevus	55	72	127
	Metastatic–primary	47	31	78

**Table 2:** The degree of miRNAs (top 10) in the miRNA-gene network.

miRNAs	Degree
hsa-miR-106b-5p	67.0
hsa-miR-27b-3p	54.0
hsa-miR-141-3p	50.0
hsa-miR-9-5p	49.0
hsa-miR-130b-3p	49.0
hsa-miR-200a-3p	42.0
hsa-miR-23b-3p	36.0
hsa-miR-200c-3p	36.0
hsa-miR-205-5p	33.0
hsa-miR-200b-3p	24.0

## Data Availability

The data supporting the findings of this study are available from the GEO database (GSE34460, GSE35579, GSE18509, and GSE24996 datasets).
